# Tooth wear as a means to quantify intra-specific variations in diet and chewing movements

**DOI:** 10.1038/srep34037

**Published:** 2016-09-23

**Authors:** Ivan Calandra, Gaëlle Labonne, Ellen Schulz-Kornas, Thomas M. Kaiser, Sophie Montuire

**Affiliations:** 1 GEGENAA – EA3795, Université de Reims Champagne-Ardenne, Reims, France; 2 Biogéosciences – UMR CNRS 6282, Université Bourgogne Franche Comté et École Pratique des Hautes Études, Dijon, France; 3 Max Planck Weizmann Center for Integrative Archaeology and Anthropology, Max Planck Institute for Evolutionary Anthropology, Leipzig, Germany; 4 Evolutionary Biology and Paleoanthropology, Center of Natural History, Hamburg, Germany; 5 Biogéosciences – UMR CNRS 6282, Université Bourgogne Franche Comté et École Pratique des Hautes Études, PSL Research University, Dijon, France

## Abstract

In mammals, tooth function, and its efficiency, depends both on the mechanical properties of the food and on chewing dynamics. These aspects have rarely been studied in combination and/or at the intra-specific level. Here we applied 3D dental surface texture analysis to a sample of field voles (*Microtus agrestis*) trapped from Finnish Lapland at different seasons and localities to test for inter-population variations. We also explored intra-individual variation in chewing dynamics by analysing two facets on the second upper molars. Our results confirm that the two localities have similar environments and that the voles feed on the same items there. On the other hand, the texture data suggest that diets are seasonally variable, probably due to varying concentrations of abrasives. Lastly, the textures on the buccal facets are more isotropic and their direction deviates more from the mesial chewing direction than the lingual facets. We interpret these results as reflecting food, rather than chewing, movements, where food particles are more guided on the lingual side of the molars. This has implications for the application of dental microwear analysis to fossils: only homologous facets can be compared, even when the molar row seems to constitute a functional unit.

In mammals, teeth are essential to fracture food particles so that enzymes in the digestive track can efficiently extract nutrients. Tooth functionality, therefore, depends on (1) the biomechanical properties of ingesta and (2) the chewing dynamics, related to chewing movements and forces. Tooth function has been approached from three complementary lines of evidence: functional morphology^1^,^2^, dietary reconstructions^3^,^4^ and reconstructions of chewing mechanics^5^,^6^. It has been shown repeatedly^7^ that tooth function cannot be reconstructed solely from the tooth morphology because the form only constraints what an animal can eat and because the actual function (what the animal ate) also depends on other ecological factors like food availability, competition and predation. Therefore, proxies independent of tooth form are required to reconstruct both diet and chewing mechanics. Intra-specific variations in tooth function remain understudied; yet, understanding how and how much tooth function varies intra-specifically, and even intra-individually, is essential to calibrate paleoenvironmental reconstructions based on dietary inferences at the inter-specific level, and to study micro- and macroevolution.

Rodents represent an important part of the fossil record due to their abundance, ubiquity and diversity throughout the Cenozoic^8^. Additionally, unlike large mammals, rodents are non-migratory and short-lived animals, which make them particularly useful in reconstructions of past climates at local and regional scales. Within rodents, Arvicolinae (voles and lemmings) constitute an excellent mammalian model group for biostratigraphy^9^ and terrestrial paleoecological and paleoenvironmental reconstructions^10^,^11^, as well as for understanding the role of development in the evolution of tooth morphology^12^,^13^. Although it is known from field observations that their diets and habitats are very diverse, arvicolines are usually regarded as grazers in open and cold paleoenvironments^14^,^15^. This lack of precision precludes detailed local paleoenvironmental reconstructions. Arvicolines have characteristic prismatic and hypsodont (high crowned) or hypselodont (hypsodont and ever-growing) molars^16^. Although the chewing dynamics of arvicolines seems to be quite simple, in which the whole flat molar row shears ingesta in mesio-distal movements^17^, the movements of food particles is still unknown. Understanding this aspect has implications for the study of vole evolution, and may also enlighten that of lagomorphs and proboscideans, all having flat molars with transversal enamel bands^16^.

Two families of proxies can be applied to fossil teeth to reconstruct intra-specific variations in tooth function: stable isotope and dental wear analyses. Stable isotopes can be measured at the individual level for seasonal and geographical variations^18^,^19^. The isotopic composition in tooth tissues reflects the chemical properties of the diet; yet, chewing aspects are neglected. Dental wear analyses also yield a signal at the individual or intra-individual (intra- and inter-tooth) level, and study the mechanical wear left by ingesta during chewing. Dental microwear patterns have a fast turnover rate, from hours to weeks (the ‘Last Supper’ effect^20^). Therefore, dental microwear analysis is a non-destructive tool that can be applied to fossil material at small temporal, geographical and individual scales to reconstruct both dietary composition and feeding biomechanics.

Microwear analysis^21^ has originally been described as a 2D semi-quantitative approach to describe wear patterns on a microscopic level in order to reconstruct diets^22^ and chewing movements based on the orientation of striations^23^,^24^. Its limits and applicability to fossil teeth became the foundation for the development of 3D approaches in the last 10–15 years, grouped under the term Dental Microwear Texture Analysis (DMTA^25^). At least three different DMTA approaches have been developed using either interferometry (Scale Sensitive Fractal Analysis, SSFA^26^), focus variation (Microtextural Analysis^27^), or disc-scanning confocal microscopy (Surface Texture Analysis, STA^28^; Fig. 1). These 3D approaches have reduced^29^, although not completely removed^30^, intra- and inter-observer errors.

Nevertheless, there are few studies on the intra-specific variations in chewing mechanics^31^,^32^ and in diet^33^,^34^ in mammals other than human populations. Moreover, the application of DMTA to rodents is still in its infancy (SSFA^34^,^35^,^36^, STA^37^). Finally, DMTA has never been applied to reconstruct chewing movements, most likely because SSFA does not compute texture direction. But in STA^28^,^38^, oriented measurements are taken to calculate the texture direction relative to the chewing force vectors.

Here, we present the first study of intra-specific variations in the tooth function of field voles based on surface texture analysis. Specifically, we test (1) whether subtle geographical and seasonal variations in the diet of voles lead to different tooth textures intra-specifically, and (2) whether texture direction and isotropy reveal variations in chewing and food movements between the buccal and lingual facets of a single molar.

## Results

The dental texture parameters of the voles from two Finnish localities Kilpisjärvi and Pallasjärvi are very similar in autumn (P > 0.1), except for the mean of the parameter Closed hill area (P_*Sha*_ = 0.046; Fig. 2 and Table 1; Supplementary Fig. S2 and Supplementary Tables S4 and S5). Therefore the general surface texture is similar but for the area of the textural hills being larger in the specimens from Kilpisjärvi. The lack of differences between the localities justifies the pooling of the samples for further analyses.

The sample size of spring individuals (n = 5) was very low. Therefore, we report only the tendency for the five parameters that seem to differ between the seasons (Fig. 2; Supplementary Fig. S3 and Supplementary Table S4). The tendency shows that the textures of voles in the spring have deeper valleys (*Sdv*), larger hills (*Shv*), higher wavelength (*Sal*), larger texture directions (*Std*), and lower heights (*Smc*) at which the areal material ratio is satisfied. This implies a coarser texture with possibly more tooth material removal (wear) in spring voles.

The lingual facet on the T3 has flatter (lower *Sal* and volume parameters *Sdv, Shv, Vm, Vmc, Vmp, Vv, Vvc, Vvv*, and higher density of peaks *Spd*) and less smooth (lower peak curvature *Spc*), although less variable (in *Sdv, Shv, Smc*), textures than the buccal facet on the T2 (Fig. 3 and Table 1; Supplementary Fig. S4 and Supplementary Tables S4 and S5). Additionally, the T3 facet has less isotropic (lower *Str*) textures than the T2 facet. The main texture directions (*Std*) on the T2 and T3 facets deviate from each other by about 30° (*Std*_meanT2_ = 110°, *Std*_meanT3_ = 79°; Supplementary Table S4) and from the mesio-distal food ingestion direction (90°).

## Discussion

The field vole (*Microtus agrestis*), as other microtine rodents, is primarily a grass-eater^39^. Its diet is centred on monocotyledonous herbs (grass and sedge), but also includes dicotyledonous herbs (forbs) and almost everything available, even animal matter^19^,^40^,^41^,^42^,^43^,^44^. It is, therefore, not surprising that its diet, as inferred from surface texture analysis, is similar at both localities studied (Fig. 2, Supplementary Fig. S2). Nevertheless, this signal is likely to be restricted to this species, and to localities similar and/or close to each other like the two localities of Pallasjärvi and Kilpisjärvi. Indeed, in both cases, voles were sampled in birch forests, where habitat structure and load of external abrasives were similar. In contrast, Butet & Delettre^39^ reviewed field studies of rodent diets and found important differences in fungi, mosses, lichens, vegetative parts of plants, bark, seeds/fruits and invertebrates consumption in the field vole between Sweden, UK and continental Europe. Such different diets would likely produce different textures. Additionally, different species may cohabit in more contrasted habitats, so that this species may avoid competition through different feeding niche displacements depending on which other species are present. For example, based on stable isotope analyses of vole teeth from Kilpisjärvi and Pallasjärvi, *Microtus agrestis* and *M. oeconomus* seem to partition food resources only in winter^19^. This relaxed competition in the winter allows them to coexist, despite the more intense interference competition in the summer^45^,^46^.

Seasonal variation seems more present than geographical variation in surface texture of Finnish voles. Spring specimens tend to have coarser textures (wider plateaus with more voluminous hills and dales: higher *Sal, Sdv, Shv*) than autumn specimens (Fig. 2). This needs to be confirmed on larger samples, especially in the spring. Field observations evidenced seasonal dietary variations in Arctic environments, where field voles differ slightly in their grass and sedge consumption (ca. 70% in autumn *vs.* 50% in spring), which they compensate mainly with forbs (ca. 15% in autumn but 40% in spring^40^,^41^,^42^,^43^,^44^). Grass and sedge contain more silica phytoliths that dicots^47^, so autumn voles would be subject to a higher concentration of internal plant abrasives that would abrade the enamel more intensively, resulting in finer textures (see hypothesis 1 in ref. 48; see also below), as seems to be the case here. Moreover, the grass and sedge bolus of autumn voles would be more resistant to deformation than that of spring voles feeding on dicots, leading to less attrition (tooth-tooth contacts) to flatten the relief in autumn (see hypothesis 3 in ref. 48). This seems to be supported by the coarser textures of spring voles.

When looking at intra-tooth variation, we have found less but more voluminous hill/dales/peak texture on the buccal facet (T2) compared to the lingual facet (T3) (Fig. 3). These results were unexpected, because it is classically assumed that the whole molar row of arvicoline rodents functions as a unit^49^, comminuting the food with a mesio-distal chewing movement in the same way elephants (with only two opposing teeth), rabbits and horses (with bucco-lingual chewing directions) do^17^. In this model, it is assumed that the prismatic molars of arvicolines would shear the food particles at every point where an enamel ridge from the lower teeth contact another from upper teeth. The chewing and food fracture mechanics were, therefore, expected to be the same at each of these contact sites.

Kaiser *et al*.^48^ proposed six hypotheses linking texture parameters to variations in ingesta and mastication. The set of hypotheses are proposed to explain the aetiology of textures, considering food material properties (concentration and size of abrasives in hypotheses 1 and 2, and resistance of the matrix in hypothesis 3), as well as chewing force and movements (hypotheses 4–6, see below). The observed intra-tooth differences in voles cannot be explained by different material properties of the ingested foods; they can only be the result of chewing mechanics, ruling out hypotheses related to ingesta properties (hypotheses 1–3). Hypotheses 4 (variations in chewing force) and 5 (variations in the horizontal component of occlusal movements) should not vary within a tooth either. Thus, only hypothesis 6 (variations in the occlusal degree of freedom, ODF) may vary in the case of voles. Kaiser *et al*.^48^ proposed that ODF is negatively related to *Spd* (density of peaks) and positively to volume parameters (*Vmc* and *Vvc*). Our results show that the T2 facet has a lower density of peak (*Spd*) and coarser texture (higher *Sal, Sdv, Shv,* all volume parameters, and higher peak curvature *Spc*) than the T3 facet (Figs 3 and 4b). Therefore, the T2 facet has a higher ODF than the T3 facet. This is consistent with the results on *Str* (texture aspect ratio), which is a measure of texture isotropy: the textures on the T2 facet are more isotropic than those on the T3 facet, although both facets have anisotropic textures (*Str* ≤ 0.05; ref. 28). This means that the food movements are less constrained on the T2 facet. As indicated by *Std* (texture direction), the T2 facet has a main direction around 110°, while the direction on T3 facet is around 80° (90° corresponds to a purely mesial direction; Fig. 4a). The skulls were always oriented similarly during scanning, so the observed variations in texture direction cannot result from variations in skull orientation during data acquisition. Therefore surface texture direction may not necessarily indicate chewing movement; instead we interpret these results as reflective of differential food movements at a microscopic scale across the enamel ridges of the tooth.

**Figure 1:**
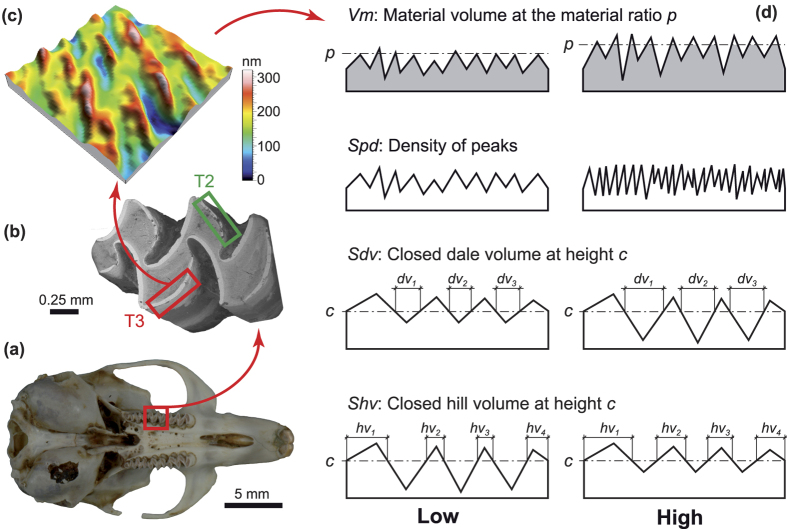
Surface texture analysis from the skull to ISO 25178-2 parameters. **(a)** Skull of *Microtus agrestis* UB-8-19 in occlusal view. (**b**) SEM photograph of the right second upper molar of specimen UB-8-28, with locations of scanned areas on the facets of the mesial enamel bands of T2 (green rectangle) and T3 (red rectangle). Note that the enamel band on the T2 of this specimen is broken, so it was not analysed (see Supplementary Table S1). **(c)** Subsurface 10 × 10 μm^2^ from the T3 facet of specimen UB-7-1. **(d)** Schematic representations of four ISO parameters: *Vm, Spd, Sdv* and *Shv*. 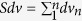 (*n* = 3 in this example) and 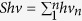 (*n* = 4 in this example).

**Figure 2:**
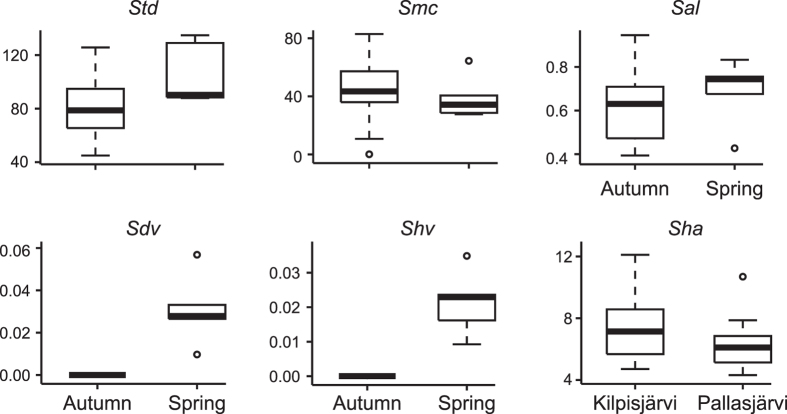
Boxplots of seasonal and geographical variations in field voles. Only the parameters showing significant differences are plotted for geographical variations (Kilpisjärvi *vs.* Pallasjärvi), while all the parameters that seem to support seasonal differences (autumn *vs.* spring) are displayed. The thick horizontal lines mark the median; the boxes enclose the first (25%) and third (75%) quartiles, i.e. the interquartile range (IQR); the whiskers (dashed lines) extend to up to 1.5 IQR; open circles represent outliers, i.e. beyond 1.5 IQR. See Supplementary Table S2 for a description of the parameters.

**Figure 3:**
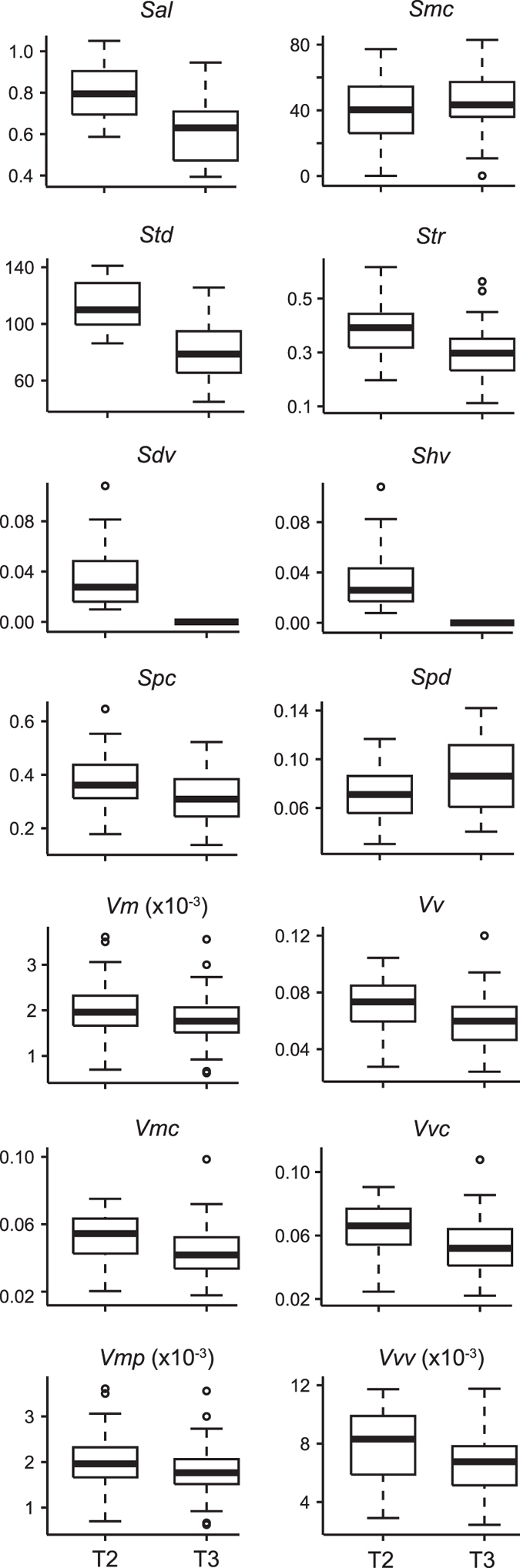
Boxplots of intra-tooth variation in field voles. Only the parameters significantly separating the tooth facets (mesial enamel bands of T2 *vs.* T3) are shown. See Fig. 2 for details of boxplots and Supplementary Table S2 for a description of the parameters.

**Figure 4:**
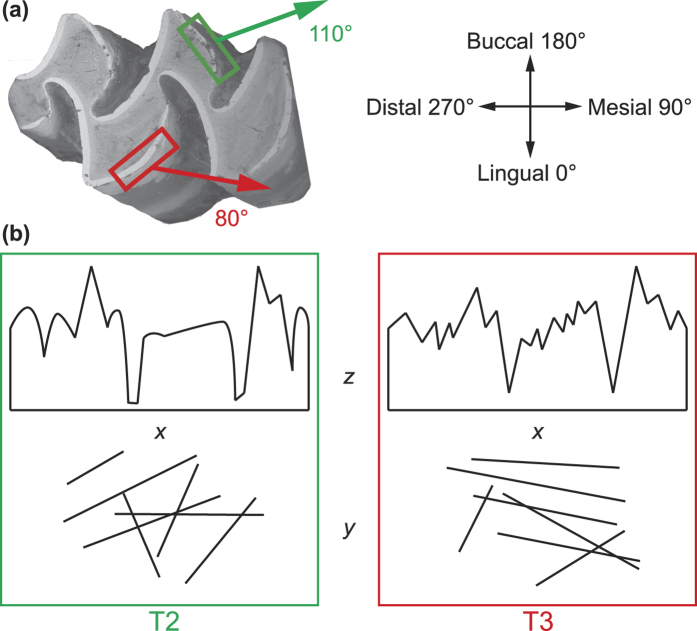
Intra-tooth variations in textures. **(a)** Texture directions (*Std*) on the T2 (green) and T3 (red) facets relative to the biological orientation. **(b)** Schematic representation of the textures on each facet. *x* and *y* represent the horizontal plane, and *z* marks the vertical axis.

To summarize, we were able to measure texture isotropy and direction (Fig. 4). Our results show that ingesta movements are quite well constrained within the mesial chewing stroke on the lingual side (*Std* ≈ 80° and *Str* ≈ 0.3), while ingesta on the buccal side are subjected to less guidance (*Std* ≈ 110° and *Str* ≈ 0.4). Because it is difficult to imagine how the chewing stroke could be differentially constrained within a single flat vole molar (Fig. 1a,b), we rather propose that it is the movements of food particles that are unevenly guided across the occlusal surface. The flat morphology is probably unable to efficiently trap particles, so when the mandible moves forward in occlusion, the broken particles would be ejected toward the cheeks (buccal side) because the tongue may prevent the particles from escaping on the lingual side. The prismatic morphology^50^, and especially the gaps between prisms, would facilitate this escape. This mechanism is consistent with the texture direction on the buccal facet (110°) deviating from the mesial orientation (90°) (Fig. 4a).

It has been shown in primates^31^ and carnivores^32^ that dietary reconstructions with DMTA should be based on the analysis of homologous facets. Our results emphasize that this is also true for arvicolines, although they do not have such contrasted functions between facets within a tooth, and probably also between teeth along the tooth row. It, therefore, raises the question of the homogeneity of tooth function along lophs in proboscideans, lagomorphs and even equids^28^.

## Conclusion

This study is the first to have addressed intra-specific variations in tooth function in terms of both food properties and chewing mechanics. For this purpose, surface texture analysis represents a powerful tool because it can detect variations at the smallest scales (intra-tooth and short-term) and also measure the direction of food movements. Voles, and rodents in general, being ubiquitous in fossil collection, such a methodology would help in local paleoenvironmental reconstructions and in micro-evolutionary studies. Yet, when applied to fossil material, any method has important limitations, and only the combination of different proxies (such as stable isotope and dental wear analyses) will allow a comprehensive view to be drawn.

## Methods

### Vole specimens

Fifty-eight field voles (*Microtus agrestis*, Arvicolinae, Rodentia) were trapped in autumn (September 2010) and spring (June 2011) at two sites in Finnish Lapland (Supplementary Fig. S1 and Supplementary Table S1). The first site, Pallasjärvi, is a boreal taiga zone. The second site, Kilpisjärvi, in the north-westernmost part of Finland, is characterized at higher altitudes by alpine tundra, with subarctic mountain birch forests at lower altitudes. At the Kilpisjärvi site, the voles were trapped in the forest habitat, which resembles the taiga of Pallasjärvi.

According to the Finnish Act on the Use of Animals for Experimental Purposes (62/2006) and a further decision by the Finnish Animal Experiment Board (16th May, 2007), the animal capture technique we used (traps that instantly kill the animal) is not considered an animal experiment and therefore requires no animal ethics license from the Finnish Animal Experiment Board. None of the captured species are included in the Red List of Finnish Species. The trapping was conducted in accordance with these guidelines.

The diet of this vole is seasonally variable and well known in the Arctic^19^,^39^,^40^,^41^,^42^,^43^,^44^. In the spring and summer, it feeds on grass/sedge (ca. 50%, up to 80%), forbs (ca. 40%) and shrubs (ca. 10%), complemented by bark and fungi (spring), and invertebrates and berries (summer). In the autumn and winter, grass/sedge (ca. 70%, up to 90%) and forbs (ca. 15%, up to 65%) constitute the main components of its diet, complemented by invertebrates and berries (in autumn), and bark and fungi (in winter).

### Preparation of vole heads

The heads of the specimens were conserved in ethanol. Then (1) the heads were boiled for 15 minutes in demineralized water mixed with sodium hypochlorite (NaClO), (2) the tissues were mechanically removed, and (3) the skulls were boiled for a further 5–10 minutes and left to dry. All dried skulls were cleaned again shortly before the analyses, in order to remove all remaining tissue and any dirt or dust deposited during storage. The skulls were (1) boiled in demineralized water for 15 min, (2) any fragments visible under the binocular of tissue were removed with a wooden toothpick, and (3) the skulls were then cleaned in an ultrasonic bath for one hour. All dried skulls are housed at the Biogéosciences Research Unit (University of Burgundy, Dijon, France).

### 3D data acquisition and analysis

The facets on the mesial enamel band of the third triangle (T3, on the lingual side of the molar) and of the second triangle (T2, on the buccal side of the molar) were scanned on the second upper molars (M^2^) of *Microtus agrestis* (Fig. 1a,b) with a high-resolution confocal disc-scanning surface measuring system (μsurf Custom, NanoFocus AG, Oberhausen, Germany, 100x lens) for surface texture analysis. The skulls were always precisely orientated mesio-distally under the objective, allowing texture directions to be recorded relative to the direction of food ingestion.

The acquired 3D surfaces were then processed with μsoft Analysis Premium v.5.1 Software (NanoFocus AG; a derivative of Mountains-Map Analysis software by DigitalSurf, Besançon, France). As the enamel bands are much thinner than the field of view, four sub-surfaces (10 × 10 μm^2^) were manually extracted along the enamel band (Fig. 1c). The area of these sub-surfaces is very small relative to the actual resolution of the scans. We are aware that this may represent a methodological limitation. For any future study, we highly recommend increasing the area of the sub-surfaces or the use of higher magnification (e.g. 150x lens). Vole teeth might be the smallest tooth size measurable with the 100x long distance lens, because it was impossible to increase the area of sub-surfaces while keeping the biological orientation of the samples. Each of these sub-surfaces was processed following Schulz *et al*.^38^: (1) the surfaces from the right molars were mirrored in *x* (to have the same orientation for all teeth), (2) levelling (least square plane by subtraction), (3) spatial filtering (denoising median 5 × 5 filter size and Gaussian 3 × 3 filter size with default cut-offs), and (4) computation of 30 ISO 25178-2 parameters^51^. These ISO parameters quantify basic geometric properties of surface textures (e.g. height, area and volume), as well as the properties of specific features (e.g. density of peaks, texture isotropy and direction) (Fig. 1d and Supplementary Table S2; see also Table 2 in ref. 38 for a complete list of ISO 25178-2 parameters and Fig. 2 in ref. 48 for schematic representations of some parameters). The eleven height parameters were not analysed because of an error during computation/export, and one parameter (*Smr*) was constant, so it was removed from subsequent analyses too; thus, 18 ISO parameters were eventually analysed. The median^26^ of the four sub-surfaces was computed for each sample and used in subsequent analyses.

### Statistics

Three datasets were analysed: (1) data on the T3 facet of autumn individuals of both localities (n_Kilpisjärvi_ = 32, n_Pallasjärvi_ = 17) to test for inter-locality differences, (2) data on the T3 facet of all specimens (localities pooled) to test for seasonal variability (n_autumn_ = 49, n_spring_ = 5), and (3) data on both T2 and T3 facets (n = 34 and 49 respectively) of all autumn individuals (localities pooled) to test for intra-tooth variation. Localities have been be pooled because no differences were detected between them (see *Results*). Due to small samples in the spring, the second dataset was only qualitatively analysed. The following statistical procedure was applied to the other two datasets. Because the goal was to understand how surface textures vary intra-specifically, rather than to separate groups based on known variations, multivariate analyses were not performed. Some ISO parameters were not normally distributed (Shapiro-Wilk tests: Supplementary Table S3), so Wilcoxon-Mann-Whitney tests were run on each parameter. Levene’s tests were also performed to test for differences in variances between groups. Adjustments of P-values for multiple comparisons were not performed to balance type I and type II errors^52^,^53^, and because only few significant differences were found (18 out of 72, see *Results*).

The open-source software package R 3.2.3 (ref. 54) was used with the following packages: car^55^, doBy^56^, R.utils^57^, readxl^58^, RSvgDevice^59^ and xlsx^60^.

## Additional Information

**How to cite this article**: Calandra, I. *et al*. Tooth wear as a means to quantify intra-specific variations in diet and chewing movements. *Sci. Rep.* **6**, 34037; doi: 10.1038/srep34037 (2016).

## Supplementary Material

Supplementary Information

## Figures and Tables

**Table 1:** Wilcoxon-Mann-Whitney tests on effects locality and facet.

Parameter	Effect Locality		Effect Facet	
	*Autumn, T3*		*Autumn, localities pooled*	
	*U*	P	*U*	P
*Sal*	295.5	0.624	1,397	**<0.001**
*Sda*	304	0.512	781	0.635
*Sdq*	244	0.567	963	0.232
*Sdr*	246	0.596	956	0.258
*Sdv*	254	0.716	1,666	**<0.001**
*Sha*	367	**0.046**	970	0.207
*Shv*	282	0.843	1,666	**<0.001**
*Smc*	292	0.685	713	0.270
*Spc*	205	0.164	1,093	**0.016**
*Spd*	197	0.117	502	**0.002**
*Std*	333	0.206	1,454	**<0.001**
*Str*	267	0.925	1,269	**<0.001**
*Vm*	211	0.206	1,046	**0.049**
*Vmc*	271	0.992	1,132	**0.005**
*Vmp*	211	0.206	1,046	**0.049**
*Vv*	273	0.992	1,125	**0.006**
*Vvc*	269	0.959	1,127	**0.006**
*Vvv*	287	0.763	1,129	**0.006**

Bold values indicate significance (P ≤ 0.05). See Supplementary Table S2 for a description of the parameters. P: P-value, *U*: Wilcoxon-Mann-Whitney test statistic.
