# Supplementary material for: Tooth wear as a means to quantify intra-specific variations in diet and chewing movements
Source: Sci Rep. 2016 Sep 23;6:34037. doi: 10.1038/srep34037 (PMC5034321; doi:10.1038/srep34037)
Supplement: Supplementary Information [file srep34037-s1.pdf]

Tooth wear as a means to quantify intra-specific variations in diet and chewing movements - *Scientific Reports 2016, 6:34037*
Ivan Calandra, Gaëlle Labonne, Ellen Schulz-Kornas, Thomas M. Kaiser & Sophie Montuire

Supplementary Table S1. Material list
(a) Parameters *Sal* to *Str*

Specimen	Locality	Season	T	*Sal*	*Sda*	*Sdq*	*Sdr*	*Sdv*	*Sha*	*Shv*	*Smc*	*Spc*	*Spd*	*Std*	*Str*
UB-07-01	Pallasjärvi	Autumn	T2	1.050	5.247	0.139	0.958	1.61E-02	4.955	1.59E-02	0.105	0.646	0.081	106.992	0.443
UB-07-01	Pallasjärvi	Autumn	T3	0.394	5.217	0.114	0.642	4.54E-11	5.199	5.10E-11	42.915	0.522	0.137	47.918	0.333
UB-07-02	Pallasjärvi	Autumn	T2	0.904	9.081	0.132	0.863	1.08E-01	10.306	8.22E-02	77.289	0.371	0.071	125.625	0.256
UB-07-02	Pallasjärvi	Autumn	T3	0.630	7.858	0.154	1.169	5.63E-11	6.230	6.60E-11	68.048	0.366	0.096	70.599	0.289
UB-07-03	Pallasjärvi	Autumn	T2	0.732	6.988	0.151	1.140	4.83E-02	6.764	5.37E-02	69.716	0.458	0.086	127.503	0.472
UB-07-03	Pallasjärvi	Autumn	T3	0.473	5.576	0.143	1.026	7.60E-11	4.886	6.89E-11	65.717	0.365	0.117	51.197	0.244
UB-07-04	Pallasjärvi	Autumn	T2	0.801	7.498	0.156	1.229	3.68E-02	8.036	4.37E-02	28.993	0.458	0.056	113.239	0.421
UB-07-04	Pallasjärvi	Autumn	T3	0.709	7.664	0.108	0.581	7.05E-11	10.691	1.02E-10	22.842	0.309	0.046	96.163	0.242
UB-07-05	Pallasjärvi	Autumn	T2	0.921	6.773	0.143	1.023	3.69E-02	6.954	1.71E-02	28.635	0.352	0.051	106.750	0.474
UB-07-05	Pallasjärvi	Autumn	T3	0.630	8.179	0.122	0.757	1.11E-10	7.716	7.95E-11	65.890	0.344	0.041	77.621	0.169
UB-07-06	Pallasjärvi	Autumn	T2	0.895	6.972	0.134	0.892	2.24E-02	6.318	1.86E-02	63.362	0.329	0.081	140.998	0.497
UB-07-06	Pallasjärvi	Autumn	T3	0.788	8.027	0.118	0.702	7.30E-11	6.849	6.88E-11	26.191	0.304	0.061	90.004	0.406
UB-07-07	Pallasjärvi	Autumn	T3	0.552	7.096	0.144	1.031	1.10E-10	5.867	7.96E-11	66.452	0.309	0.071	76.739	0.258
UB-07-08	Pallasjärvi	Autumn	T2	0.611	6.628	0.149	1.112	3.13E-02	5.252	1.98E-02	26.605	0.527	0.066	103.495	0.318
UB-07-08	Pallasjärvi	Autumn	T3	0.788	6.157	0.171	1.459	6.42E-11	7.035	9.68E-11	0.106	0.490	0.101	106.237	0.351
UB-07-09	Pallasjärvi	Autumn	T3	0.709	6.360	0.092	0.417	4.44E-11	6.462	4.64E-11	44.400	0.214	0.091	65.322	0.389
UB-07-10	Pallasjärvi	Autumn	T3	0.630	7.175	0.151	1.142	1.25E-10	5.374	6.95E-11	75.417	0.504	0.142	70.513	0.297
UB-07-15	Pallasjärvi	Autumn	T2	0.944	5.828	0.100	0.502	1.35E-02	6.613	2.67E-02	51.693	0.264	0.071	108.373	0.479
UB-07-15	Pallasjärvi	Autumn	T3	0.394	3.712	0.063	0.198	1.84E-11	4.319	1.56E-11	23.506	0.230	0.137	55.724	0.267
UB-07-16	Pallasjärvi	Autumn	T2	0.751	8.374	0.140	0.983	7.96E-02	6.345	3.09E-02	71.122	0.397	0.086	133.367	0.516
UB-07-16	Pallasjärvi	Autumn	T3	0.473	6.772	0.163	1.320	1.14E-10	6.190	5.85E-11	35.442	0.309	0.086	65.494	0.141
UB-07-17	Pallasjärvi	Autumn	T3	0.473	5.730	0.122	0.747	6.38E-11	6.105	5.55E-11	51.670	0.337	0.112	74.523	0.220
UB-07-19	Pallasjärvi	Autumn	T2	0.738	8.125	0.114	0.653	1.59E-02	5.681	3.32E-02	68.903	0.338	0.086	86.257	0.373
UB-07-19	Pallasjärvi	Autumn	T3	0.788	7.431	0.072	0.262	5.25E-11	7.870	5.15E-11	38.690	0.203	0.061	45.016	0.323
UB-07-20	Pallasjärvi	Autumn	T2	0.865	6.951	0.096	0.463	1.85E-02	9.935	1.63E-02	24.081	0.294	0.056	88.249	0.432
UB-07-20	Pallasjärvi	Autumn	T3	0.552	5.242	0.097	0.465	2.74E-11	5.134	3.12E-11	37.606	0.270	0.101	124.104	0.400
UB-07-21	Pallasjärvi	Autumn	T2	0.904	6.215	0.086	0.374	2.76E-02	5.140	7.73E-03	44.311	0.329	0.051	128.874	0.317
UB-07-21	Pallasjärvi	Autumn	T3	0.630	6.417	0.142	1.074	7.31E-11	4.874	5.52E-11	22.556	0.497	0.122	76.752	0.450
UB-07-22	Pallasjärvi	Autumn	T2	0.954	5.948	0.132	0.865	9.89E-03	13.425	1.55E-02	44.342	0.344	0.051	128.998	0.277
UB-07-23	Pallasjärvi	Autumn	T2	0.838	5.507	0.096	0.455	1.69E-02	5.754	2.51E-02	51.377	0.341	0.081	93.754	0.430
UB-07-23	Pallasjärvi	Autumn	T3	0.473	5.029	0.111	0.620	3.83E-11	5.110	3.84E-11	50.628	0.391	0.112	111.615	0.300

Supplementary Table S1 (*continued*)
(a) Parameters *Sal* to *Str* (continued)

Specimen	Locality	Season	T	*Sal*	*Sda*	*Sdq*	*Sdr*	*Sdv*	*Sha*	*Shv*	*Smc*	*Spc*	*Spd*	*Std*	*Str*
UB-08-01	Kilpisjärvi	Autumn	T2	0.679	4.987	0.062	0.195	1.22E-02	6.740	1.67E-02	26.119	0.178	0.071	89.879	0.314
UB-08-01	Kilpisjärvi	Autumn	T3	0.394	5.115	0.053	0.142	1.59E-11	5.696	1.50E-11	24.365	0.138	0.117	78.718	0.112
UB-08-02	Kilpisjärvi	Autumn	T2	0.816	6.110	0.117	0.688	2.08E-02	7.436	2.92E-02	64.145	0.395	0.066	107.493	0.442
UB-08-02	Kilpisjärvi	Autumn	T3	0.788	6.107	0.090	0.406	3.40E-11	5.038	3.59E-11	46.841	0.266	0.101	109.390	0.528
UB-08-03	Kilpisjärvi	Autumn	T3	0.946	9.761	0.165	1.373	1.31E-10	9.549	8.59E-11	0.135	0.436	0.086	45.028	0.233
UB-08-04	Kilpisjärvi	Autumn	T2	0.587	5.661	0.098	0.487	1.04E-02	7.056	4.68E-02	35.035	0.260	0.081	108.375	0.318
UB-08-04	Kilpisjärvi	Autumn	T3	0.552	5.991	0.120	0.736	5.70E-11	8.724	6.40E-11	55.498	0.222	0.051	94.786	0.227
UB-08-06	Kilpisjärvi	Autumn	T2	0.822	5.636	0.131	0.870	2.65E-02	7.345	1.94E-02	49.266	0.231	0.030	115.990	0.350
UB-08-07	Kilpisjärvi	Autumn	T2	0.769	6.464	0.145	1.047	5.02E-02	7.057	3.46E-02	33.111	0.413	0.071	92.499	0.385
UB-08-07	Kilpisjärvi	Autumn	T3	0.630	6.799	0.085	0.360	4.28E-11	8.077	4.45E-11	41.159	0.186	0.071	92.234	0.317
UB-08-08	Kilpisjärvi	Autumn	T2	0.672	7.406	0.113	0.636	3.05E-02	6.146	8.05E-03	58.117	0.371	0.091	111.504	0.300
UB-08-08	Kilpisjärvi	Autumn	T3	0.552	5.699	0.140	0.977	5.75E-11	4.708	4.71E-11	63.783	0.383	0.127	44.986	0.333
UB-08-09	Kilpisjärvi	Autumn	T3	0.630	6.117	0.112	0.659	6.14E-11	6.889	3.44E-11	43.394	0.376	0.081	89.985	0.233
UB-08-11	Kilpisjärvi	Autumn	T3	0.630	8.158	0.081	0.331	6.12E-11	9.899	7.70E-11	45.763	0.192	0.041	59.336	0.310
UB-08-14	Kilpisjärvi	Autumn	T2	0.606	6.202	0.082	0.334	2.63E-02	5.034	9.19E-03	36.245	0.303	0.066	94.125	0.297
UB-08-14	Kilpisjärvi	Autumn	T3	0.473	6.657	0.126	0.809	4.76E-11	5.686	5.91E-11	67.246	0.324	0.091	83.478	0.182
UB-08-16	Kilpisjärvi	Autumn	T2	0.861	6.610	0.172	1.480	5.32E-02	10.332	2.27E-02	0.103	0.348	0.046	129.001	0.406
UB-08-17	Kilpisjärvi	Autumn	T3	0.630	7.412	0.154	1.181	8.53E-11	11.481	1.36E-10	39.038	0.278	0.041	76.565	0.203
UB-08-19	Kilpisjärvi	Autumn	T2	0.640	5.091	0.180	1.620	4.48E-02	7.726	4.32E-02	0.080	0.553	0.076	130.621	0.348
UB-08-19	Kilpisjärvi	Autumn	T3	0.552	5.638	0.114	0.645	4.77E-11	5.025	4.07E-11	44.904	0.378	0.137	111.415	0.437
UB-08-20	Kilpisjärvi	Autumn	T3	0.630	10.469	0.120	0.721	1.57E-10	9.288	6.81E-11	57.116	0.210	0.051	72.382	0.167
UB-08-22	Kilpisjärvi	Autumn	T2	0.788	6.553	0.140	0.971	6.71E-02	6.014	2.18E-02	64.816	0.381	0.091	99.497	0.434
UB-08-22	Kilpisjärvi	Autumn	T3	0.473	5.734	0.141	0.985	5.51E-11	5.437	5.67E-11	65.311	0.405	0.117	63.497	0.333
UB-08-23	Kilpisjärvi	Autumn	T2	1.043	7.564	0.120	0.720	4.43E-02	7.219	1.85E-02	0.087	0.323	0.066	107.378	0.617
UB-08-23	Kilpisjärvi	Autumn	T3	0.709	7.518	0.115	0.660	6.03E-11	8.166	6.16E-11	57.519	0.391	0.071	85.616	0.344
UB-08-24	Kilpisjärvi	Autumn	T2	0.785	8.173	0.178	1.580	6.82E-02	7.952	8.24E-02	0.116	0.541	0.086	132.249	0.496
UB-08-24	Kilpisjärvi	Autumn	T3	0.788	8.150	0.151	1.131	1.17E-10	7.561	9.23E-11	77.178	0.368	0.091	83.474	0.347
UB-08-25	Kilpisjärvi	Autumn	T3	0.630	5.674	0.084	0.354	3.18E-11	5.669	2.79E-11	36.015	0.291	0.096	123.125	0.364
UB-08-28	Kilpisjärvi	Autumn	T3	0.709	7.767	0.157	1.249	9.31E-11	5.889	6.47E-11	57.354	0.307	0.071	95.955	0.213
UB-08-29	Kilpisjärvi	Autumn	T3	0.630	6.108	0.160	1.271	7.10E-11	6.594	8.84E-11	30.871	0.409	0.127	81.493	0.358
UB-08-30	Kilpisjärvi	Autumn	T3	0.473	7.435	0.174	1.506	8.20E-11	7.497	1.04E-10	82.894	0.483	0.086	81.891	0.177
UB-08-33	Kilpisjärvi	Autumn	T3	0.788	8.234	0.114	0.649	7.20E-11	7.943	7.86E-11	46.578	0.254	0.056	104.910	0.380
UB-08-34	Kilpisjärvi	Autumn	T2	0.600	4.752	0.103	0.531	2.21E-02	5.853	1.71E-02	49.218	0.400	0.117	139.368	0.322
UB-08-34	Kilpisjärvi	Autumn	T3	0.473	8.004	0.138	1.025	1.23E-10	7.146	8.39E-11	10.737	0.389	0.051	85.636	0.292

Supplementary Table S1 (*continued*)
(a) Parameters *Sal* to *Str* (continued)

Specimen	Locality	Season	T	*Sal*	*Sda*	*Sdq*	*Sdr*	*Sdv*	*Sha*	*Shv*	*Smc*	*Spc*	*Spd*	*Std*	*Str*
UB-08-36	Kilpisjärvi	Autumn	T2	0.897	6.429	0.118	0.710	2.75E-02	6.702	2.16E-02	45.557	0.458	0.086	128.757	0.489
UB-08-37	Kilpisjärvi	Autumn	T2	0.766	10.942	0.161	1.293	8.14E-02	12.081	1.08E-01	0.092	0.478	0.030	116.125	0.361
UB-08-37	Kilpisjärvi	Autumn	T3	0.788	7.899	0.109	0.593	6.75E-11	9.280	1.22E-10	15.581	0.238	0.056	78.772	0.367
UB-08-38	Kilpisjärvi	Autumn	T2	0.934	7.545	0.161	1.284	5.69E-02	9.749	5.46E-02	0.087	0.393	0.066	112.496	0.399
UB-08-38	Kilpisjärvi	Autumn	T3	0.552	6.832	0.162	1.293	7.79E-11	12.111	2.19E-10	33.151	0.304	0.056	51.192	0.254
UB-08-39	Kilpisjärvi	Autumn	T3	0.630	5.907	0.083	0.347	5.65E-11	8.007	4.95E-11	40.250	0.177	0.086	59.857	0.354
UB-08-40	Kilpisjärvi	Autumn	T3	0.473	4.185	0.116	0.667	2.94E-11	4.816	3.41E-11	40.572	0.323	0.112	78.725	0.303
UB-08-41	Kilpisjärvi	Autumn	T2	0.695	4.676	0.125	0.783	1.41E-02	10.486	4.17E-02	54.453	0.288	0.056	96.495	0.197
UB-08-41	Kilpisjärvi	Autumn	T3	0.473	5.863	0.127	0.804	6.85E-11	4.994	3.36E-11	52.215	0.417	0.096	85.606	0.261
UB-08-42	Kilpisjärvi	Autumn	T2	0.941	6.206	0.152	1.167	2.81E-02	7.392	3.59E-02	30.655	0.312	0.051	90.117	0.340
UB-08-42	Kilpisjärvi	Autumn	T3	0.552	8.341	0.110	0.613	5.07E-11	8.429	1.08E-10	57.239	0.244	0.076	57.353	0.272
UB-08-44	Kilpisjärvi	Autumn	T2	0.620	5.394	0.140	0.983	1.60E-02	6.114	3.20E-02	54.075	0.437	0.086	132.129	0.299
UB-08-44	Kilpisjärvi	Autumn	T3	0.709	4.837	0.088	0.383	3.39E-11	7.143	5.11E-11	41.405	0.204	0.101	120.657	0.563
UB-08-46	Kilpisjärvi	Autumn	T2	0.787	5.289	0.094	0.461	1.59E-02	9.058	4.36E-02	33.702	0.229	0.056	107.123	0.401
UB-08-46	Kilpisjärvi	Autumn	T3	0.630	8.075	0.114	0.654	7.46E-11	11.180	1.10E-10	48.007	0.334	0.056	76.764	0.282
UB-08-47	Kilpisjärvi	Autumn	T3	0.552	6.586	0.095	0.454	6.18E-11	6.258	5.65E-11	42.958	0.239	0.081	125.666	0.297
UB-08-48	Kilpisjärvi	Autumn	T3	0.473	5.033	0.109	0.590	3.15E-11	6.418	3.09E-11	40.681	0.274	0.076	114.517	0.233
UB-08-50	Kilpisjärvi	Autumn	T3	0.709	5.708	0.083	0.344	4.25E-11	5.337	4.07E-11	41.663	0.258	0.081	67.833	0.343
UB-22-01	Kilpisjärvi	Spring	T3	0.833	9.518	0.120	0.719	5.68E-02	6.531	1.62E-02	64.433	0.341	0.112	128.996	0.252
UB-22-02	Kilpisjärvi	Spring	T3	0.756	5.982	0.142	1.005	3.32E-02	8.136	3.49E-02	27.645	0.433	0.081	88.370	0.391
UB-22-03	Kilpisjärvi	Spring	T3	0.744	7.016	0.091	0.406	2.78E-02	7.874	2.29E-02	40.572	0.215	0.076	90.245	0.338
UB-23-04	Pallasjärvi	Spring	T3	0.427	4.189	0.079	0.312	9.70E-03	4.282	9.25E-03	28.625	0.393	0.142	134.751	0.258
UB-23-05	Pallasjärvi	Spring	T3	0.676	9.913	0.168	1.426	2.66E-02	6.240	2.36E-02	34.257	0.482	0.076	87.884	0.145

Supplementary Table S1 (*continued*)
(b) Volume parameters

Specimen	Locality	Season	T	*Vm*	*Vmc*	*Vmp*	*Vv*	*Vvc*	*Vvv*
UB-07-01	Pallasjärvi	Autumn	T2	0.003609	0.067106	0.003609	0.094662	0.085594	0.008304
UB-07-01	Pallasjärvi	Autumn	T3	0.001919	0.030557	0.001919	0.044548	0.041084	0.004270
UB-07-02	Pallasjärvi	Autumn	T2	0.001959	0.063793	0.001959	0.087903	0.079217	0.008686
UB-07-02	Pallasjärvi	Autumn	T3	0.002001	0.057833	0.002001	0.076387	0.068495	0.009176
UB-07-03	Pallasjärvi	Autumn	T2	0.003503	0.053907	0.003503	0.083838	0.076910	0.006928
UB-07-03	Pallasjärvi	Autumn	T3	0.001528	0.045661	0.001528	0.067431	0.061141	0.006767
UB-07-04	Pallasjärvi	Autumn	T2	0.002297	0.064189	0.002297	0.083431	0.073745	0.009686
UB-07-04	Pallasjärvi	Autumn	T3	0.002069	0.041768	0.002069	0.059038	0.051637	0.007403
UB-07-05	Pallasjärvi	Autumn	T2	0.002674	0.062253	0.002674	0.084821	0.074239	0.009965
UB-07-05	Pallasjärvi	Autumn	T3	0.001921	0.046673	0.001921	0.067917	0.059366	0.007209
UB-07-06	Pallasjärvi	Autumn	T2	0.001752	0.055618	0.001752	0.077335	0.069314	0.007284
UB-07-06	Pallasjärvi	Autumn	T3	0.002175	0.046779	0.002175	0.060801	0.054123	0.006678
UB-07-07	Pallasjärvi	Autumn	T3	0.001902	0.052267	0.001902	0.068204	0.060758	0.007866
UB-07-08	Pallasjärvi	Autumn	T2	0.002439	0.047154	0.002439	0.068515	0.059706	0.008320
UB-07-08	Pallasjärvi	Autumn	T3	0.002730	0.061986	0.002730	0.090749	0.081547	0.009884
UB-07-09	Pallasjärvi	Autumn	T3	0.001580	0.031951	0.001580	0.046521	0.040893	0.004972
UB-07-10	Pallasjärvi	Autumn	T3	0.002210	0.057415	0.002210	0.078909	0.071762	0.007473
UB-07-15	Pallasjärvi	Autumn	T2	0.001631	0.043324	0.001631	0.059382	0.053703	0.005756
UB-07-15	Pallasjärvi	Autumn	T3	0.000665	0.017859	0.000665	0.024155	0.022113	0.002444
UB-07-16	Pallasjärvi	Autumn	T2	0.002335	0.055468	0.002335	0.075353	0.066320	0.008347
UB-07-16	Pallasjärvi	Autumn	T3	0.002303	0.059867	0.002303	0.078698	0.070774	0.008123
UB-07-17	Pallasjärvi	Autumn	T3	0.001849	0.036608	0.001849	0.053348	0.047057	0.006974
UB-07-19	Pallasjärvi	Autumn	T2	0.002070	0.046913	0.002070	0.071319	0.065813	0.005849
UB-07-19	Pallasjärvi	Autumn	T3	0.001391	0.033674	0.001391	0.042475	0.038157	0.004489
UB-07-20	Pallasjärvi	Autumn	T2	0.001277	0.034676	0.001277	0.053384	0.046749	0.008500
UB-07-20	Pallasjärvi	Autumn	T3	0.001249	0.026725	0.001249	0.038859	0.034407	0.004386
UB-07-21	Pallasjärvi	Autumn	T2	0.001557	0.039916	0.001557	0.054793	0.049464	0.005328
UB-07-21	Pallasjärvi	Autumn	T3	0.002351	0.050763	0.002351	0.068590	0.060432	0.007339
UB-07-22	Pallasjärvi	Autumn	T2	0.002284	0.061732	0.002284	0.091875	0.083642	0.009675
UB-07-23	Pallasjärvi	Autumn	T2	0.001960	0.042143	0.001960	0.060960	0.054271	0.006689
UB-07-23	Pallasjärvi	Autumn	T3	0.001772	0.036435	0.001772	0.052411	0.046755	0.005147

Supplementary Table S1 (*continued*)
(b) Volume parameters (*continued*)

Specimen	Locality	Season	T	*Vm*	*Vmc*	*Vmp*	*Vv*	*Vvc*	*Vvv*
UB-08-01	Kilpisjärvi	Autumn	T2	0.000700	0.020283	0.000700	0.027614	0.024637	0.002906
UB-08-01	Kilpisjärvi	Autumn	T3	0.000626	0.017868	0.000626	0.024960	0.022411	0.002549
UB-08-02	Kilpisjärvi	Autumn	T2	0.002164	0.042617	0.002164	0.065815	0.056981	0.008131
UB-08-02	Kilpisjärvi	Autumn	T3	0.001558	0.035437	0.001558	0.048652	0.044083	0.004437
UB-08-03	Kilpisjärvi	Autumn	T3	0.003559	0.098559	0.003559	0.119961	0.107777	0.010329
UB-08-04	Kilpisjärvi	Autumn	T2	0.001363	0.034929	0.001363	0.045808	0.040804	0.005004
UB-08-04	Kilpisjärvi	Autumn	T3	0.001334	0.041194	0.001334	0.056967	0.048717	0.007703
UB-08-06	Kilpisjärvi	Autumn	T2	0.001788	0.055094	0.001788	0.065534	0.056702	0.008655
UB-08-07	Kilpisjärvi	Autumn	T2	0.002278	0.063401	0.002278	0.078166	0.068097	0.010069
UB-08-07	Kilpisjärvi	Autumn	T3	0.001568	0.030233	0.001568	0.042874	0.038638	0.005357
UB-08-08	Kilpisjärvi	Autumn	T2	0.001839	0.043509	0.001839	0.059518	0.054603	0.005003
UB-08-08	Kilpisjärvi	Autumn	T3	0.001820	0.047313	0.001820	0.065944	0.059104	0.006839
UB-08-09	Kilpisjärvi	Autumn	T3	0.001622	0.037697	0.001622	0.054119	0.048150	0.006040
UB-08-11	Kilpisjärvi	Autumn	T3	0.001291	0.034366	0.001291	0.047237	0.041766	0.004980
UB-08-14	Kilpisjärvi	Autumn	T2	0.001093	0.026596	0.001093	0.037212	0.033022	0.004725
UB-08-14	Kilpisjärvi	Autumn	T3	0.002064	0.048426	0.002064	0.069239	0.064190	0.005802
UB-08-16	Kilpisjärvi	Autumn	T2	0.001817	0.074313	0.001817	0.104320	0.090550	0.011106
UB-08-17	Kilpisjärvi	Autumn	T3	0.002520	0.071928	0.002520	0.094041	0.085496	0.009552
UB-08-19	Kilpisjärvi	Autumn	T2	0.002322	0.060442	0.002322	0.082693	0.073538	0.009895
UB-08-19	Kilpisjärvi	Autumn	T3	0.001655	0.037528	0.001655	0.051841	0.046355	0.005687
UB-08-20	Kilpisjärvi	Autumn	T3	0.001515	0.042889	0.001515	0.061432	0.051803	0.008343
UB-08-22	Kilpisjärvi	Autumn	T2	0.001890	0.056339	0.001890	0.078090	0.067152	0.009397
UB-08-22	Kilpisjärvi	Autumn	T3	0.002056	0.053009	0.002056	0.069805	0.062057	0.007829
UB-08-23	Kilpisjärvi	Autumn	T2	0.002105	0.056730	0.002105	0.079343	0.069371	0.009972
UB-08-23	Kilpisjärvi	Autumn	T3	0.001799	0.041350	0.001799	0.059341	0.051999	0.006409
UB-08-24	Kilpisjärvi	Autumn	T2	0.002955	0.075080	0.002955	0.100355	0.090161	0.010195
UB-08-24	Kilpisjärvi	Autumn	T3	0.002547	0.064615	0.002547	0.092490	0.082099	0.009992
UB-08-25	Kilpisjärvi	Autumn	T3	0.001245	0.025497	0.001245	0.037283	0.031894	0.005007
UB-08-28	Kilpisjärvi	Autumn	T3	0.003000	0.061796	0.003000	0.082670	0.073255	0.008622
UB-08-29	Kilpisjärvi	Autumn	T3	0.002091	0.061700	0.002091	0.086356	0.076046	0.009207
UB-08-30	Kilpisjärvi	Autumn	T3	0.002369	0.067189	0.002369	0.089201	0.080781	0.006840
UB-08-33	Kilpisjärvi	Autumn	T3	0.001961	0.040218	0.001961	0.065516	0.057914	0.006932
UB-08-34	Kilpisjärvi	Autumn	T2	0.001561	0.034781	0.001561	0.050774	0.045708	0.005066
UB-08-34	Kilpisjärvi	Autumn	T3	0.001629	0.049803	0.001629	0.071684	0.064102	0.007582

Supplementary Table S1 (*continued*)
(b) Volume parameters (*continued*)

Specimen	Locality	Season	T	*Vm*	*Vmc*	*Vmp*	*Vv*	*Vvc*	*Vvv*
UB-08-36	Kilpisjärvi	Autumn	T2	0.001782	0.048062	0.001782	0.062082	0.054682	0.007400
UB-08-37	Kilpisjärvi	Autumn	T2	0.003060	0.070178	0.003060	0.095267	0.082698	0.011729
UB-08-37	Kilpisjärvi	Autumn	T3	0.001764	0.039648	0.001764	0.059785	0.050593	0.009191
UB-08-38	Kilpisjärvi	Autumn	T2	0.002502	0.065418	0.002502	0.088969	0.077047	0.010988
UB-08-38	Kilpisjärvi	Autumn	T3	0.001955	0.055348	0.001955	0.078723	0.066156	0.011758
UB-08-39	Kilpisjärvi	Autumn	T3	0.001101	0.030549	0.001101	0.041406	0.034983	0.006423
UB-08-40	Kilpisjärvi	Autumn	T3	0.001398	0.035173	0.001398	0.047398	0.042336	0.005153
UB-08-41	Kilpisjärvi	Autumn	T2	0.001666	0.049355	0.001666	0.065661	0.058105	0.007556
UB-08-41	Kilpisjärvi	Autumn	T3	0.001595	0.044114	0.001595	0.060182	0.053880	0.006340
UB-08-42	Kilpisjärvi	Autumn	T2	0.002135	0.067590	0.002135	0.087743	0.077012	0.010731
UB-08-42	Kilpisjärvi	Autumn	T3	0.001260	0.046527	0.001260	0.058495	0.052303	0.005960
UB-08-44	Kilpisjärvi	Autumn	T2	0.001663	0.052288	0.001663	0.068690	0.061072	0.007618
UB-08-44	Kilpisjärvi	Autumn	T3	0.000921	0.031596	0.000921	0.042706	0.037754	0.004952
UB-08-46	Kilpisjärvi	Autumn	T2	0.001301	0.035453	0.001301	0.051773	0.045893	0.005881
UB-08-46	Kilpisjärvi	Autumn	T3	0.001637	0.046938	0.001637	0.064401	0.055790	0.007040
UB-08-47	Kilpisjärvi	Autumn	T3	0.001590	0.032055	0.001590	0.044988	0.041041	0.004794
UB-08-48	Kilpisjärvi	Autumn	T3	0.001098	0.033277	0.001098	0.041687	0.036769	0.005475
UB-08-50	Kilpisjärvi	Autumn	T3	0.001559	0.029355	0.001559	0.043099	0.039153	0.004750
UB-22-01	Kilpisjärvi	Spring	T3	0.001709	0.043639	0.001709	0.066984	0.058894	0.008019
UB-22-02	Kilpisjärvi	Spring	T3	0.002391	0.050315	0.002391	0.070594	0.061132	0.009463
UB-22-03	Kilpisjärvi	Spring	T3	0.001736	0.031184	0.001736	0.046982	0.040504	0.004941
UB-23-04	Pallasjärvi	Spring	T3	0.001247	0.020771	0.001247	0.029931	0.027166	0.002765
UB-23-05	Pallasjärvi	Spring	T3	0.002578	0.066314	0.002578	0.092327	0.082697	0.009630

All specimens are housed at the University of Burgundy, abbreviation 'UB'. See Supplementary Table S2 for a description of the parameters. T: triangle.

Supplementary Table S2. Description of the 18 ISO 25178-2 parameters used.

Parameter	Description	Unit
Bearing ratio		
Smc	Inverse areal material ration (*p*=10%)	µm
Feature		
Sda	Closed dale area	$µm^2$
Sdv	Closed dale volume	$µm^3$
Sha	Closed hill area	$µm^2$
Shv	Closed hill volume	$µm^3$
Spc	Arithmetic mean peak curvature	$µm^{-1}$
Spd	Density of peaks	$µm^{-2}$
Hybrid		
Sdq	Root mean square gradient	no unit
Sdr	Developed interfacial area ratio	%
Spatial		
Sal	Auto-correlation length (*s*=0.2)	µm
Std	Texture direction	°
Str	Texture aspect ration (*s*=0.2)	no unit
Volume		
Vm	Material volume at a given material ratio (*p*=10%)	$µm^3/µm^2$
Vmc	Material volume of the core at given material ratio (*p*=10%, *q*=80%)	$µm^3/µm^2$
Vmp	Material volume of peaks (*p*=10%)	$µm^3/µm^2$
Vv	Void volume at a given material ratio (*p*=10%)	$µm^3/µm^2$
Vvc	Void volume of the core at given material ratio (*p*=10%, *q*=80%)	$µm^3/µm^2$
Vvv	Void volume of the valleys at a given material ratio (*q*=80%)	$µm^3/µm^2$

Supplementary Table S3. Shapiro-Wilk normality tests on residuals.

	**Effect Locality**		**Effect Facet**	
	Autumn, T3		*Autumn (localities pooled)*	
Parameter	*W*	P	*W*	P
Sal	0.958	0.078	0.969	**0.041**
Sda	0.972	0.295	0.969	**0.044**
Sdq	0.975	0.382	0.982	0.282
Sdr	0.958	0.081	0.971	0.059
Sdv	0.939	**0.014**	0.734	**<0.001**
Sha	0.938	**0.012**	0.913	**<0.001**
Shv	0.882	**<0.001**	0.668	**<0.001**
Smc	0.977	0.445	0.968	**0.037**
Spc	0.971	0.254	0.982	0.302
Spd	0.977	0.431	0.981	0.276
Std	0.967	0.186	0.980	0.214
Str	0.979	0.524	0.982	0.311
Vm	0.962	0.115	0.971	0.059
Vmc	0.945	**0.022**	0.974	0.087
Vmp	0.962	0.115	0.971	0.059
Vv	0.971	0.273	0.989	0.691
Vvc	0.971	0.256	0.988	0.667
Vvv	0.982	0.639	0.993	0.954

Bold values indicate significance ($P \leq 0.05$), i.e. non-normal distribution. See Supplementary Table S2 for a description of the parameters. P: P-value, *W*: test statistic.

Supplementary Table S4. Descriptive statistics.

		Effect Locality		Effect Facet		Effect Season	
		Autumn, T3		*Autumn (localities pooled)*		*T3 (localities pooled)*	
		Kilpisjärvi	Pallasjärvi	T2	T3	Autumn	Spring
	n	32	17	34	49	49	5
Sal	Mean	0.613	0.593	0.800	0.606	0.606	0.687
	Median	0.630	0.630	0.795	0.630	0.630	0.744
	SD	0.125	0.134	0.128	0.127	0.127	0.156
	SEM	0.022	0.032	0.022	0.018	0.018	0.070
Sda	Mean	6.807	6.450	6.583	6.683	6.683	7.324
	Median	6.621	6.417	6.447	6.586	6.586	7.016
	SD	1.431	1.245	1.331	1.367	1.367	2.410
	SEM	0.253	0.302	0.228	0.195	0.195	1.078
Sdq	Mean	0.118	0.123	0.128	0.120	0.120	0.120
	Median	0.115	0.122	0.132	0.116	0.116	0.120
	SD	0.030	0.031	0.029	0.030	0.030	0.036
	SEM	0.005	0.007	0.005	0.004	0.004	0.016
Sdr	Mean	0.747	0.801	0.863	0.766	0.766	0.774
	Median	0.659	0.747	0.868	0.667	0.667	0.719
	SD	0.357	0.368	0.362	0.358	0.358	0.455
	SEM	0.063	0.089	0.062	0.051	0.051	0.204
Sdv	Mean	6.56E-11	6.84E-11	3.52E-02	6.65E-11	6.65E-11	3.08E-02
	Median	6.08E-11	6.42E-11	2.76E-02	6.14E-11	6.14E-11	2.78E-02
	SD	3.14E-11	3.12E-11	2.37E-02	3.11E-11	3.11E-11	1.70E-02
	SEM	5.56E-12	7.57E-12	4.07E-03	4.44E-12	4.44E-12	7.60E-03
Sha	Mean	7.373	6.230	7.499	6.976	6.976	6.612
	Median	7.144	6.105	7.005	6.462	6.462	6.531
	SD	2.041	1.533	2.054	1.943	1.943	1.540
	SEM	0.361	0.372	0.352	0.278	0.278	0.689
Shv	Mean	6.94E-11	6.08E-11	3.27E-02	6.64E-11	6.64E-11	2.14E-02
	Median	6.03E-11	5.85E-11	2.59E-02	5.91E-11	5.91E-11	2.29E-02
	SD	4.04E-11	2.21E-11	2.25E-02	3.51E-11	3.51E-11	9.54E-03
	SEM	7.13E-12	5.35E-12	3.86E-03	5.01E-12	5.01E-12	4.26E-03
Smc	Mean	45.232	43.416	37.812	44.602	44.602	39.106
	Median	44.149	42.915	40.278	43.394	43.394	34.257
	SD	17.556	20.664	24.080	18.497	18.497	15.069
	SEM	3.103	5.012	4.130	2.642	2.642	6.739
Spc	Mean	0.303	0.351	0.375	0.320	0.320	0.373
	Median	0.297	0.337	0.361	0.309	0.309	0.393
	SD	0.087	0.102	0.101	0.094	0.094	0.102
	SEM	0.015	0.025	0.017	0.013	0.013	0.046
Spd	Mean	0.082	0.096	0.070	0.087	0.087	0.097
	Median	0.081	0.101	0.071	0.086	0.086	0.081
	SD	0.026	0.032	0.019	0.029	0.029	0.029
	SEM	0.005	0.008	0.003	0.004	0.004	0.013

Supplementary Table S4 *(continued)*

		**Effect Locality**		**Effect Facet**		**Effect Season**	
		Autumn, T3		*Autumn (localities pooled)*		*T3 (localities pooled)*	
		Kilpisjärvi	Pallasjärvi	T2	T3	Autumn	Spring
Std	Mean	83.776	76.797	112.756	81.355	81.355	106.049
	Median	82.683	74.523	109.939	78.725	78.725	90.245
	SD	21.836	22.574	16.176	22.115	22.115	23.678
	SEM	3.860	5.475	2.774	3.159	3.159	10.589
Str	Mean	0.301	0.299	0.389	0.300	0.300	0.277
	Median	0.300	0.297	0.392	0.297	0.297	0.258
	SD	0.097	0.085	0.089	0.092	0.092	0.094
	SEM	0.017	0.021	0.015	0.013	0.013	0.042
Vm	Mean	0.001741	0.001860	0.002039	0.001782	0.001782	0.001932
	Median	0.001626	0.001919	0.001959	0.001764	0.001764	0.001736
	SD	0.000593	0.000481	0.000632	0.000555	0.000555	0.000544
	SEM	0.000105	0.000117	0.000108	0.000079	0.000079	0.000243
Vmc	Mean	0.044787	0.043225	0.052078	0.044245	0.044245	0.042445
	Median	0.041272	0.045661	0.054501	0.041768	0.041768	0.043639
	SD	0.016080	0.012746	0.013645	0.014890	0.014890	0.017533
	SEM	0.002843	0.003091	0.002340	0.002127	0.002127	0.007841
Vmp	Mean	0.001741	0.001860	0.002039	0.001782	0.001782	0.001932
	Median	0.001626	0.001919	0.001959	0.001764	0.001764	0.001736
	SD	0.000593	0.000481	0.000632	0.000555	0.000555	0.000544
	SEM	0.000105	0.000117	0.000108	0.000079	0.000079	0.000243
Vv	Mean	0.061703	0.059944	0.071853	0.061092	0.061092	0.061364
	Median	0.059563	0.060801	0.073336	0.059785	0.059785	0.066984
	SD	0.020181	0.017217	0.018135	0.019041	0.019041	0.023824
	SEM	0.003568	0.004176	0.003110	0.002720	0.002720	0.010654
Vvc	Mean	0.054669	0.053559	0.063721	0.054284	0.054284	0.054079
	Median	0.051901	0.054123	0.066066	0.051999	0.051999	0.058894
	SD	0.018191	0.015463	0.016019	0.017138	0.017138	0.021216
	SEM	0.003216	0.003750	0.002747	0.002448	0.002448	0.009488
Vvv	Mean	0.006809	0.006506	0.007981	0.006703	0.006703	0.006964
	Median	0.006416	0.006974	0.008312	0.006767	0.006767	0.008019
	SD	0.002024	0.001945	0.002189	0.001982	0.001982	0.003008
	SEM	0.000358	0.000472	0.000375	0.000283	0.000283	0.001345

See Supplementary Table S2 for a description of the parameters. n: sample size, SD: standard deviation, SEM: standard error of the mean.

Supplementary Table S5. Levene's tests on effects locality and facet.

	**Effect Locality** *Autumn, T3*			**Effect Facet** *Autumn (localities pooled)*		
Parameter	*df*	*F*	P	*df*	*F*	P
Sal	1, 47	0.409	0.526	1, 81	0.045	0.832
Sda	1, 47	0.420	0.520	1, 81	0.488	0.487
Sdq	1, 47	0.049	0.826	1, 81	0.035	0.851
Sdr	1, 47	0.108	0.744	1, 81	0.005	0.946
Sdv	1, 47	0.070	0.793	1, 81	46.781	**<0.001**
Sha	1, 47	2.759	0.103	1, 81	0.028	0.868
Shv	1, 47	2.245	0.141	1, 81	39.617	**<0.001**
Smc	1, 47	1.210	0.277	1, 81	4.525	**0.036**
Spc	1, 47	0.088	0.768	1, 81	0.002	0.962
Spd	1, 47	0.686	0.412	1, 81	7.845	**0.006**
Std	1, 47	0.004	0.947	1, 81	1.903	0.171
Str	1, 47	0.164	0.688	1, 81	0.028	0.867
Vm	1, 47	0.314	0.578	1, 81	0.440	0.509
Vmc	1, 47	0.124	0.727	1, 81	0.004	0.948
Vmp	1, 47	0.314	0.578	1, 81	0.440	0.509
Vv	1, 47	0.152	0.699	1, 81	0.001	0.978
Vvc	1, 47	0.122	0.728	1, 81	0.013	0.910
Vvv	1, 47	0.052	0.821	1, 81	0.604	0.439

Bold values indicate significance (P ≤ 0.05). *df*: degrees of freedom, *F*: Levene's test statistic, P: P-value. See Supplementary Table S2 for a description of the parameters.

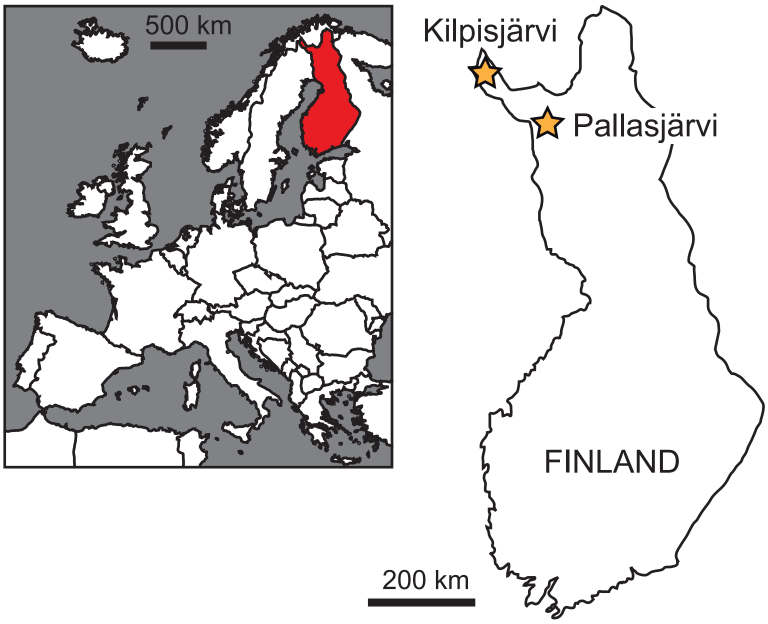

Supplementary Figure S1. Trapping localities. Maps modified with permission from http://www.histgeo.ac-aix-marseille.fr

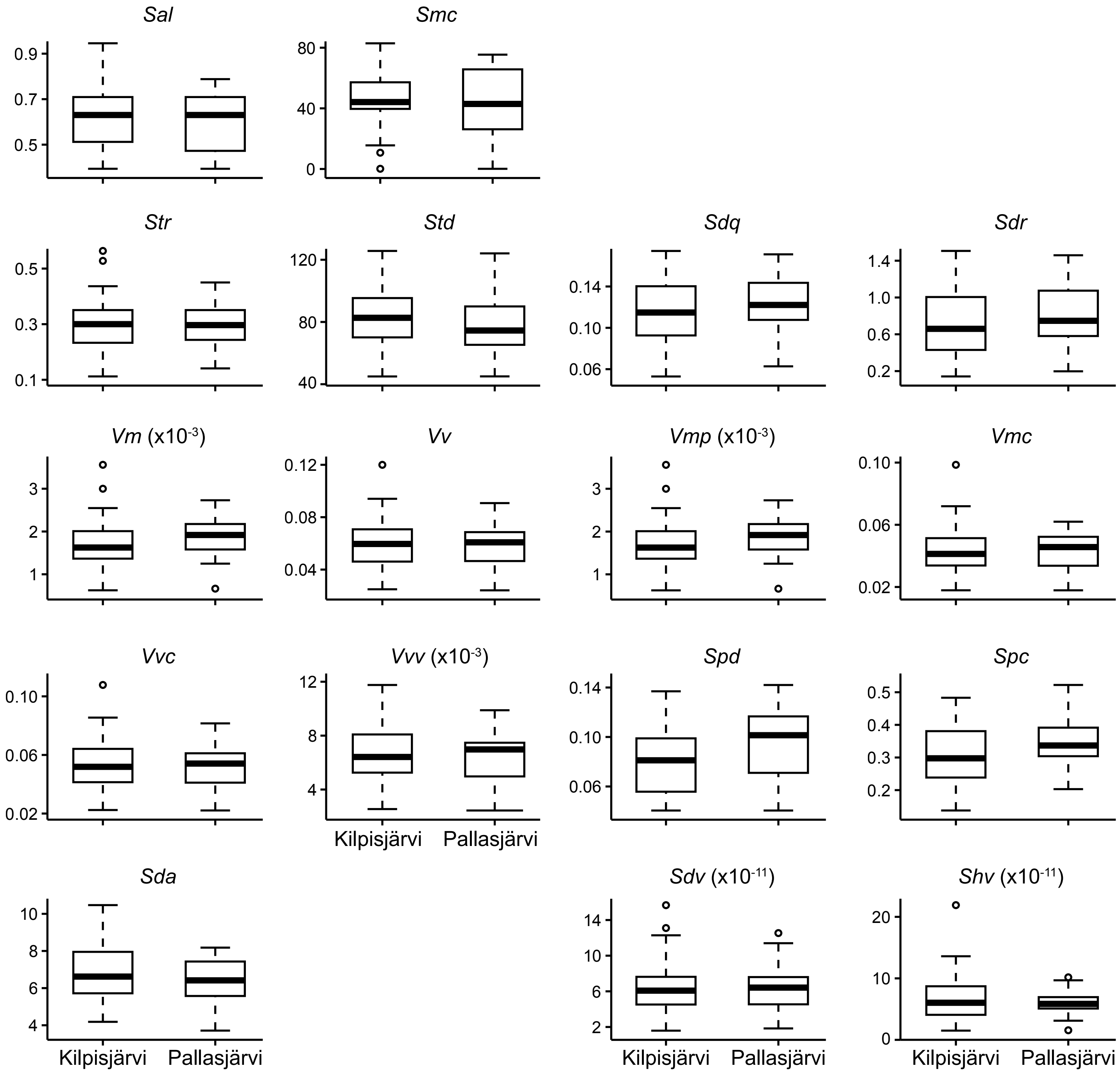

Supplementary Figure S2. Boxplots of geographical variations in field voles. Plots of the parameters not appearing in Fig. 2. See Fig. 2 for details of boxplots. See Supplementary Table S2 for a description of the parameters.

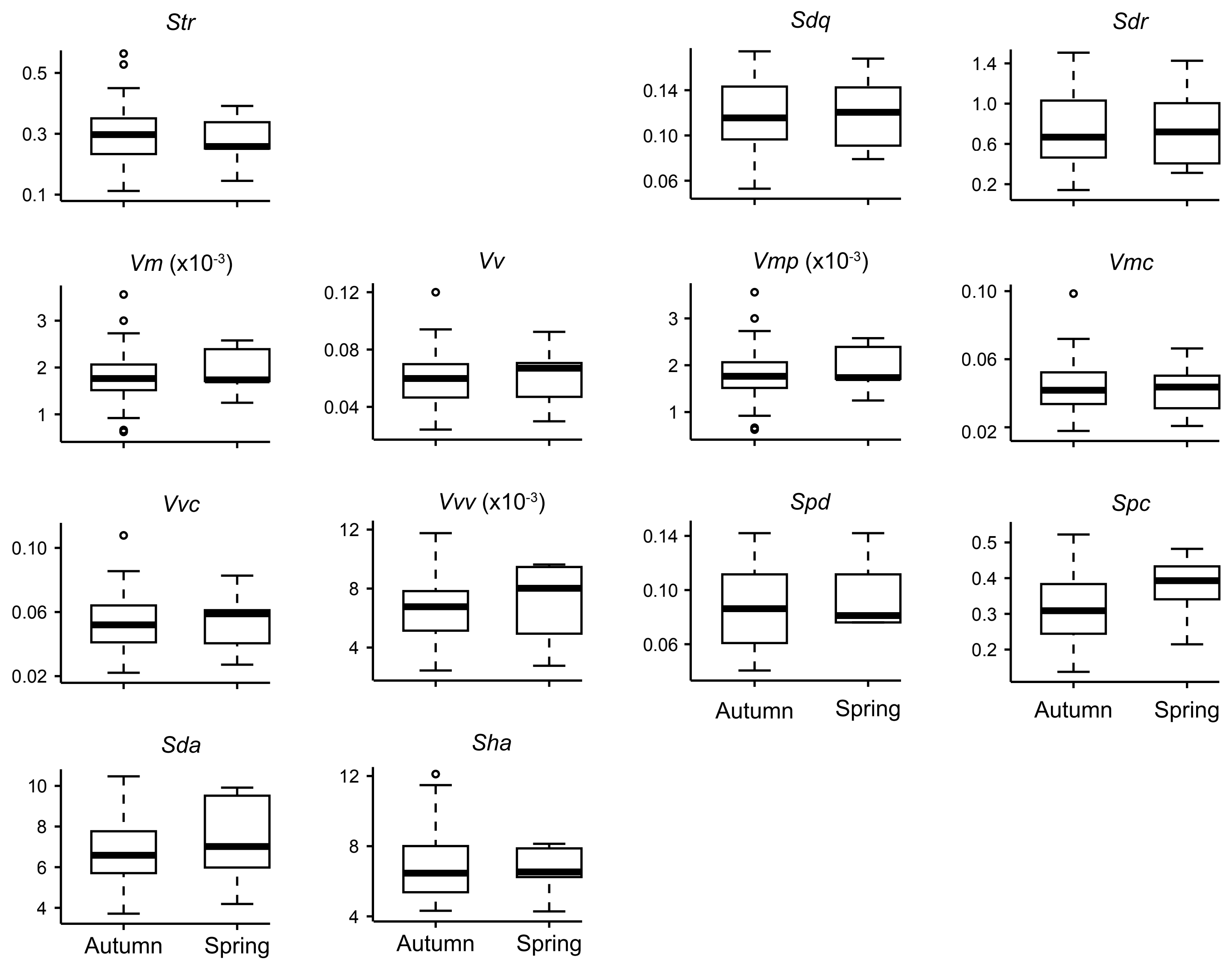

Supplementary Figure S3. Boxplots of seasonal variations in field voles. Plots of the parameters not appearing in Fig. 2. See Fig. 2 for details of boxplots. See Supplementary Table S2 for a description of the parameters.

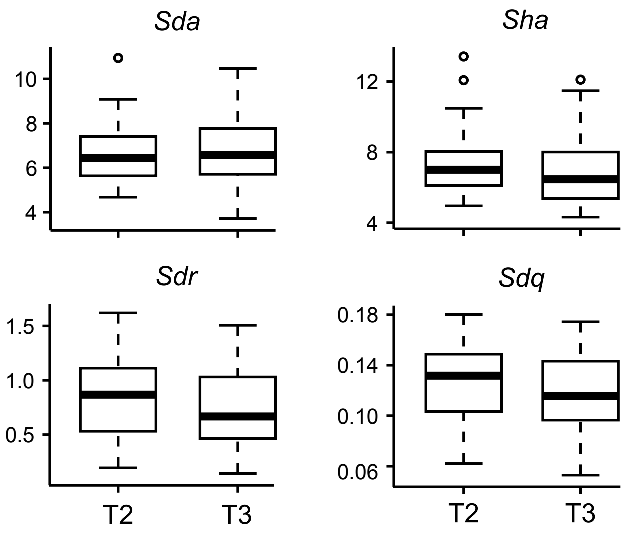

Supplementary Figure S4. Boxplots of intra-tooth variations in field voles: T2 *vs.* T3 facets. Plots of the parameters not appearing in Fig. 3. See Fig. 2 for details of boxplots. See Supplementary Table S2 for a description of the parameters.
